# Adaptable Immunofluorescence Protocol for Muscle Fiber Typing in FFPE Human and Mouse Skeletal Muscle and Intact Mouse Hindlimbs

**DOI:** 10.1002/cpz1.70246

**Published:** 2025-11-03

**Authors:** Connor Thomas, Lainey M. Hibbard, Kenneth E. White, Steven S. Welc

**Affiliations:** ^1^ Department of Anatomy, Cell Biology, & Physiology Indiana University School of Medicine, Indianapolis Indianapolis; ^2^ Department of Medical and Molecular Genetics Indiana University School of Medicine, Indianapolis Indianapolis; ^3^ Indiana Center for Musculoskeletal Health Indiana University School of Medicine, Indianapolis Indianapolis

**Keywords:** formalin‐fixed paraffin‐embedded, immunofluorescence, myosin heavy chain, skeletal muscle fiber type

## Abstract

Skeletal muscle fiber type composition affects muscle function, metabolism, and disease vulnerability. In addition, muscle fiber type analysis informs disease diagnosis and underlying pathophysiology. Multiple methodologies can be used to assess muscle fiber type; however, immunofluorescence (IF) for myosin heavy chain (MyHC) isoforms is the most widely used modern approach due to its relative ease, time‐effectiveness, single‐cell resolution, and capacity to preserve spatial positioning within the native tissue architecture. Here, we present a protocol for IF for MyHC labeling on formalin‐fixed paraffin‐embedded (FFPE) mouse and human muscle sections. We then describe a modified procedure for fiber type analysis of the intact mouse lower hindlimb, enabling high‐throughput muscle composition and morphological analysis across distinct muscles on a single tissue section. Traditionally, IF labeling for MyHC isoforms required fresh tissue flash‐frozen in liquid nitrogen–cooled isopentane, which, while effective, presents challenges for sample processing and preservation, long‐term storage, transport, and biosafety. Comparatively, embedding tissue in paraffin after formalin fixation streamlines clinical workflows, preserves morphology, improves long‐term sample stability, and simplifies sample storage and transport. Furthermore, FFPE effectively inactivates most infectious agents, which can be retained in frozen sections. Thus, FFPE samples are typically safe for standard laboratory handling and are not classified as biohazardous. This approach can be adapted for use with a range of downstream applications, including integration of fiber type analysis with emerging next‐generation techniques that favor FFPE samples. In sum, this method offers a robust alternative to traditional fresh‐frozen protocols and allows for simultaneous fiber type analysis across multiple muscle tissues. © 2025 The Author(s). Current Protocols published by Wiley Periodicals LLC.

**Basic Protocol**: Multiplex Immunofluorescence for MyHC Labeling in FFPE Skeletal Muscle Tissues

**Alternate Protocol 1**: Immunofluorescence for MyHC 2x Labeling in FFPE Skeletal Muscle Tissues

**Alternate Protocol 2**: Multiplex Immunofluorescence for MyHC Labeling in FFPE Whole Hindlimb Sections

## Introduction

Skeletal muscle is a heterogeneous tissue composed of muscle fiber types with distinct contractile, metabolic, and physiological properties. Muscle fiber types can broadly be categorized into type 1, type 2a, type 2x, and type 2b fibers. Type 1 fibers are slow‐twitch, highly oxidative, and resistant to fatigue. Type 2a fibers are fast‐twitch but predominantly rely on oxidative metabolism and provide intermediate endurance and force. Type 2x fibers are fast‐twitch, primarily glycolytic, generate high force, and fatigue quickly. Type 2b fibers, which are present in mice but absent in humans, are the fastest contracting, most glycolytic, and produce maximal force with low fatigue resistance (Burkholder et al., 1994; Pette & Staron, 2001; Schiaffino & Reggiani, 2011). Fiber type composition influences muscle performance, energy metabolism, and susceptibility to injury or disease (LeBrasseur et al., 2011; Wang & Pessin, 2013). In both research and clinical contexts, accurate determination of muscle composition and fiber type distribution provides valuable insight into muscle function, adaptive remodeling, and the pathophysiology of neuromuscular disorders.

Multiple approaches have been developed to assess muscle fiber types. Early histochemical methods, such as myofibrillar ATPase staining, enabled classification based on enzymatic activity but required labor‐intensive pH pre‐incubations, were prone to technical variation, offered limited resolution of fiber sub‐types, and were limited in their capacity for integration with other multi‐marker labeling approaches (Hintz et al., 1984). Discoveries that distinct myosin isoforms are differentially expressed among muscle fiber types led to the emergence of MyHC detection as a more accurate and versatile method for fiber classification. Gel electrophoresis of single isolated muscle fibers is the most accurate method for quantitative molecular characterization of MyHC isoforms (Murach et al., 2019). However, this method is technical, low throughput, labor‐intensive, and spatial or morphological relationships are lost with mechanical separation and homogenization. The development and validation of monoclonal antibodies against specific MyHC isoforms, together with the accessibility of standardized IF reagents and fluorescence microscopy, have driven the widespread adoption of MyHC IF for muscle composition analysis. MyHC IF offers specific, reproducible, multiplexed, and morphology‐preserving analysis at single‐cell resolution, which is unmatched by other methods.

Conventionally, MyHC IF is most commonly performed on fresh‐frozen muscle sections. However, fresh‐frozen tissue preparation presents numerous challenges. Cryopreservation requires rapid freezing in liquid nitrogen‐cooled isopentane, specialized equipment, and experienced handling. Frozen tissue must be stored at ultra‐low temperatures, is vulnerable to degradation during long‐term storage or transport, and retains infectious agents, necessitating additional biosafety measures. Practical considerations, such as the need for immediate freezing in liquid nitrogen‐cooled isopentane to preserve tissue morphology and prevent freeze artifacts, limit the compatibility of cryopreservation with certain clinical and research applications.

To expand the ability to perform MyHC IF and analyze muscle composition in different clinical and research settings, we adapted the MyHC IF approach for use with formalin‐fixed paraffin‐embedded (FFPE) human and mouse skeletal muscle. FFPE is a common histological preparation in which tissue samples are chemically fixed and then embedded in paraffin wax for stabilization and sectioning. FFPE provides a practical and accessible alternative to cryopreservation. Formalin fixation crosslinks proteins to preserve tissue morphology and inactivates most pathogens, enhancing biosafety, while paraffin embedding enables long‐term storage, supporting longitudinal and comparative studies using both new and existing archived biobank samples (Möller et al., 2015). FFPE preparations can be easily integrated into routine clinical tissue sampling, avoid the logistical challenges of immediate freezing in the clinic, and are especially well‐suited for archival tissue collections and transporting tissues across multi‐site workflows (Engel & Moore, 2011). Furthermore, the ability to perform MyHC IF on FFPE sections complements next‐generation technologies, including spatial transcriptomics or other methods that are best suited for FFPE samples. Lastly, FFPE allows MyHC IF to be performed on transverse sections of the whole hindlimb, which is difficult to achieve with cryopreservation due to technical challenges associated with cutting mineralized tissue and freezing and handling of large tissue blocks.

Here, we describe three FFPE‐based multiplex IF protocols for MyHC isoforms. **Basic Protocol** describes a standard method for simultaneous detection of MyHC isoforms in FFPE muscle sections, using antibodies against MyHC 1, MyHC 2a, and MyHC 2b in mouse, and against MyHC 1 and MyHC 2a in human. Type 2x fibers are identified by the absence of immunoreactive labeling with these antibodies. This protocol includes steps for tissue fixation, deparaffinization, antigen retrieval, and antibody labeling. **Alternate Protocol** **1** details a specialized procedure optimized for the use of an antibody against MyHC 2x for direct detection of type 2x fibers that requires an alternative antigen retrieval process not compatible with immunolabeling of other MyHC isoforms. **Alternate Protocol** **2** presents a modified approach for IF fiber typing in the intact mouse lower hindlimb, enabling high‐throughput analysis of composition and morphology across multiple muscles of the intact lower mouse hindlimb on a single section. Basic Protocol is ideal for broad fiber type profiling, Alternate Protocol 1 is suited for targeted analysis of type 2x fibers, and Alternate Protocol 2 facilitates comparative multi‐tissue analysis *in situ*.

*NOTE*: All protocols involving animals must be reviewed and approved by the appropriate Animal Care and Use Committee and must follow regulations for the care and use of laboratory animals. Appropriate informed consent is necessary for obtaining and use of human study material.

## MULTIPLEX IMMUNOFLUORESCENCE FOR MyHC LABELING IN FFPE SKELETAL MUSCLE TISSUES

This protocol identifies muscle fiber types by multiplex IF labeling on FFPE skeletal muscle sections based on distinct MyHC isoform expression. Antibodies against MyHC 1 (type 1 fibers), MyHC 2a (type 2a fibers), and MyHC 2b (type 2b fibers) are used to label fiber types, while the absence of these markers infers type 2x fibers. An antibody against laminin, a basement membrane protein, is used to visualize muscle fiber profiles. The procedure involves fixation, deparaffinization, antigen retrieval, and antibody labeling. Laminin will appear in the DAPI channel; type 1 fibers in the Cy5 channel; type 2a fibers in the GFP channel; and type 2b fibers in the Texas Red channel. Type 2x fibers remain unlabeled and thus appear dark within the tissue. This protocol allows for highly specific and spatially preserved identification of muscle fiber types at single‐fiber resolution suitable for quantitative analysis.

### Materials

Isoflurane (Pivetal, cat. no. 78949580 or equivalent from an approved institutional or veterinary drug distributor)
10% Neutral Buffered Formalin (Fisherbran,d cat. no. 11002205 or equivalent)
Xylenes Histological Grade (Fisher Chemical, cat. no. BPX3P1GAL or equivalent)
200‐Proof ethanol (Decon Labs, cat. no. 04355223 or equivalent)
Ethanol solutions: 95%, 70%, 50% (see recipe)
Paraffin (Leica Biosystems, cat. no. 39601006 or equivalent)
1× PBS (see recipe)
1× PBS containing 0.25% Triton‐X100 (see recipe)
Tris‐EDTA Buffer pH = 9.0 (see recipe)
Blocking Buffer (see recipe)
Mouse IgG2b anti‐Bovine Myosin Heavy Chain Type 1 (Developmental Studies Hybridoma Bank, cat. no. BA‐D5)
Mouse IgG1 anti‐Bovine Myosin Heavy Chain Type 2a (Developmental Studies Hybridoma Bank, cat. no. SC‐71)
Mouse IgM anti‐Bovine Myosin Heavy Chain Type 2b33 (Developmental Studies Hybridoma Bank, cat. no. BF‐F3)
Rabbit anti‐Human Laminin Subunit Alpha 1 (Sigma Aldrich, cat. no. L9393)
Goat anti‐Mouse IgG1 Alexa Fluor 488 (Invitrogen, cat. no. A21121)
Goat anti‐Mouse IgG2b Alexa Fluor 647 (Invitrogen, cat. no. A21242)
Goat anti‐Mouse IgM Alexa Fluor 594 (Invitrogen, cat. no. A21044)
Goat anti‐Rabbit IgG Alexa Fluor 350 (Invitrogen, cat. no. A11046)
TrueBlack Lipofuscin Autofluorescence Quencher (Biotium, cat. no. 23007)
Prolong Gold Antifade Mountant (Invitrogen, cat. no. P36934)

Surgical Gauze (Henry Schein, cat. no. 1408896 or equivalent)
Dissection Board (Fisherbrand, cat. no. 09‐002‐16 or equivalent)
Fine Surgical Scissors (Fine Science Tools, cat. no. 14060‐09 or equivalent)
Blunt End Forceps (Graham‐Field, cat. no. 19‐027502 or equivalent)
Blunt Scissors (Fisherbrand, cat. no. 138062 or equivalent)
Fine Point forceps (Fisherbrand, cat. no. 12‐000‐126 or equivalent)
Minutien Pins (Fine Science Tools, cat. no. 26002‐20)
Cork sheet (Grainger, cat. no. 4NLW6)
Tissue Embedding Cassette (Simport, cat. no. M490 or equivalent)
Metal Base Mold (Tissue‐Tek, cat. no. 4132 or equivalent)
Tissue Processor (Leica Biosystems ASP 300s or equivalent)
Embedding Station (Sakura Finetek Tissue‐Tek TEC 6 or equivalent)
Rotary Microtome (Leica Biosystems RM2125RTS or equivalent)
Disposable Steel Blades (Leica Biosystems, cat. no. 14035843488 or equivalent)
Slide Dryer (Triangle Biomedical Sciences, cat. no. 15‐184‐42 or equivalent)
Water Bath (Van Waters and Rogers, cat. no. 396324 or equivalent)
Microscope Slides (Fisher Anatomical, cat. no. 22034979 or equivalent)
Chemical Fume Hood (Fisher Hamilton or equivalent)
Instant Pot (Instant Pot Model Pro 60 or equivalent pressure cooker)
Cover Glass (Globe Scientific, cat. no. 10027‐469 or equivalent)
Polypropylene Coplin Staining Jar (Bel‐Art, cat. no. F44208‐1000)
Glass Coplin Staining Jar (Wheaton, cat. no. 900620)
EasyDip Slide Staining Kit (Simport, cat. no. M906‐12AS)
Hydrophobic Barrier Pen (Newcomer Supply, cat. no. 6505 or equivalent)
1000‐µl pipette tips (Fisherbrand, cat. no. 2707402 or equivalent)
200‐µl pipette tips (Fisherbrand, cat. no. 2707418 or equivalent)
10‐µl pipette tips (Fisherbrand, cat. no. 2707440 or equivalent)
P1000 pipette (Gilson, cat. no. F123602 or equivalent)
P200 pipette (Gilson, cat. no. F123601 or equivalent)
P10 pipette (Gilson, cat. no. F144055M or equivalent)
15‐ml Conical tube (Corning, cat. no. 352096 or equivalent)
Microscope (Zeiss AxioObserver 7 or equivalent epifluorescence microscope compatible with DAPI, GFP, Texas Red, and Cy5 filter sets)
  All animal procedures described in this protocol were performed in accordance with guidelines approved by the Institutional Animal Care and Use Committee (IACUC) at Indiana University School of Medicine (protocol #22045, approval date: 3 August 2022). Researchers intending to use this protocol must obtain prior approval from IACUC or their equivalent regulatory body before performing the following procedures.

#### Formalin Fixation and Paraffin Embedding

**Figure 1:**
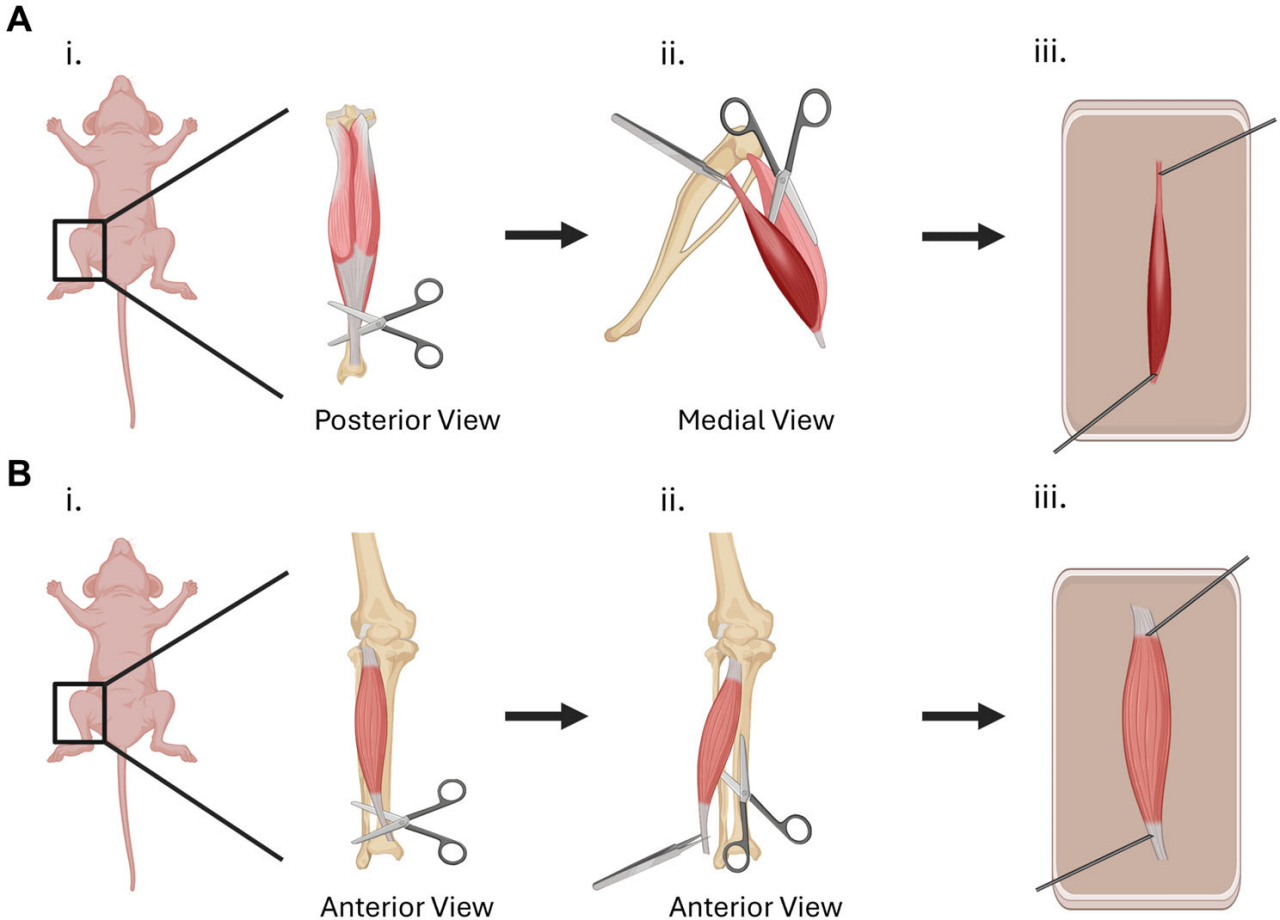
Mouse soleus and TA dissections: (**A**) Schematic overview of a mouse soleus muscle dissection. Steps include: (i) Removing the skin from the mouse to expose the lower hind limb and making a transverse cut across the Achilles tendon, (ii) Separating the posterior muscle compartment from the surrounding connective tissue and bone and subsequently removing the soleus from the gastrocnemius, (iii) Pinning the isolated soleus at resting length to a cork board for formalin fixation. (**B**) Schematic overview of a mouse TA muscle dissection. Steps include: (i) Removing the skin from the mouse to expose the lower hindlimb and making a transverse cut across the distal tendon of the TA, (ii) Isolating the TA by performing longitudinal cuts along the lateral border of the tibia and lateral border of the TA, (iii) Pinning the isolated TA at resting length to a cork board for formalin fixation.

1 Euthanize the mouse using anesthetic overdose with isoflurane (or according to standard lab protocol). Perform a secondary physical method of euthanasia, such as cervical dislocation.
  Perform procedures involving isoflurane in a chemical fume hood. For isoflurane‐based euthanasia, saturate surgical gauze with isoflurane and place in a 15‐ml conical tube. Cap the conical tube and allow the isoflurane to vaporize for 5 min. Scruff the mouse and place its nose inside the 15‐ml conical tube, maintaining this position until at least 1 min after respirations have ceased. Perform a secondary physical method of euthanasia.
2 Dissect the muscle of interest from the mouse immediately after euthanasia.
  For dissecting the mouse soleus, position the mouse prone and saturate the mouse with 70% ethanol. Remove the skin to expose the lower hindlimb. Identify and make a transverse cut across the Achilles tendon at the posterior ankle (Fig. 1Ai). Free the gastrocnemius and soleus from the lower hindlimb by making longitudinal cuts from distal to proximal to separate the muscles from surrounding connective tissue. Once the gastrocnemius and soleus are released, identify the soleus, which lies deep to the gastrocnemius and appears dark red due to its higher myoglobin content. Using fine forceps and scissors, carefully cut the origin tendon and use surgical scissors to carefully remove the soleus from the gastrocnemius (Fig. 1Aii).
  For dissecting the mouse tibialis anterior (TA), position the mouse supine and saturate with 70% ethanol. Remove the skin to expose the lower hindlimb. Identify the TA, which is the prominent muscle of the anterior compartment of the lower leg located lateral to the tibia. Using fine scissors, carefully make a transverse cut across the distal tendon of the TA at the anterior ankle (Fig. 1Bi). To free the muscle, first make a longitudinal cut along the lateral border of the tibia. Then, make a longitudinal cut on the lateral border of the TA to separate from adjacent musculature (Fig. 1Bii).
  For human skeletal muscle biopsies or surgical specimens, proceed directly to neutral buffered formalin fixation following excision (Basic Protocol Step 4).

3 Use minutien pins to secure the tendons of the muscle to a cork board. Adjust the spacing between the pinned tendons so that the muscle is held just beyond slack length, applying slight tension to maintain physiological resting length (Fig. 1Aiii, 1Biii).
  Optional step: pinning muscles at resting length is an optional step that can prevent stretching or contraction artifacts that cause variation in quantitative morphological analysis.
4 Submerge the muscle in 10% neutral buffered formalin and incubate for 24 hr at 4°C.
5 Rinse the muscle with 1× PBS three times for 5 min each to remove residual fixative.
6 Transfer the muscle to 70% ethanol for at least 24 hr at 4°C to begin dehydration.
7 Transfer the muscle to a tissue‐embedding cassette.
8 Perform dehydration, paraffin embedding, and sectioning according to your lab's or histology facility's standard protocol. Cut sections at 5‐µm thickness.
  Example dehydration, paraffin embedding, and sectioning procedure:

#### Dehydration

9 Place the tissue‐embedding cassette containing the muscle into a tissue processor to perform dehydration and clearing.
10 On the tissue processor, set and perform the following program:
11 Wash with 70% ethanol for 45 min at room temperature (RT),
12 Second wash with 70% ethanol for 45 min at RT,
13 Wash with 80% ethanol for 45 min at RT,
14 Second wash with 80% ethanol for 45 min at RT,
15 Wash with 100% ethanol for 45 min at RT,
16 Second wash with 100% ethanol for 45 min at RT,
17 Clear with xylene for 45 min at RT,
18 Second clearance with xylene for 45 min at RT,
19 Infiltrate with melted paraffin at 60°C for 60 min,
20 Second infiltration with paraffin at 60°C for 60 min,
21 Third infiltration with paraffin at 60°C for 60 min.
22 Once complete, remove tissue‐embedding cassette containing the muscle from the tissue processor.

#### Paraffin Embedding

23 Place a metal base mold into the embedding station and fill with melted paraffin maintained at 56–57°C.
24 Place the muscle into the metal base mold, orienting it so that transverse or cross sections will be obtained when sectioning.
25 Once the muscle is properly positioned, place the tissue cassette on top of the paraffin wax. Allow the paraffin block to harden completely at RT.

#### Sectioning

26 Place the paraffin block containing the muscle onto a rotary microtome equipped with disposable steel blades.
27 Cut transverse sections at 5‐µm thickness.
28 Float sections on a heated water bath set to 40–45°C to flatten the tissue sections and then collect them onto a microscope slide.
29 Allow the tissue sections to dry overnight at 37°C in a slide dryer. After drying, store at 4°C until proceeding with the remainder of the protocol.
  To achieve consistent orientation and comparable sections across samples, carefully maintain the anatomical direction and position of each muscle throughout embedding and sectioning. Place the muscles in the same anatomical orientation and location within the cassette and paraffin block, and mark the proximal end of each paraffin block with a notch to indicate orientation. When sectioning, trim excess paraffin until the tissue is exposed. Then, section the muscle at a consistent depth relative to the tendon, targeting the muscle mid‐belly so that all sections are collected from the same location within the tissue.

#### Deparaffinization and Rehydration

30 Immerse slides with tissue sections in a glass Coplin jar filled with fresh xylene. Incubate for 10 min. Transfer slides into a second glass Coplin jar containing fresh xylene and incubate for an additional 10 min.
  Perform xylene steps under a chemical fume hood.
31 Place the slides in an EasyDip Slide Staining jar containing 100% ethanol. Incubate for 10 min. Transfer the slides to a second staining jar containing fresh 100% ethanol and incubate for an additional 10 min.
32 Place the slides in an EasyDip Slide Staining jar containing 95% ethanol. Incubate for 5 min. Transfer the slides to a second staining jar containing fresh 95% ethanol and incubate for an additional 5 min.
33 Place the slides in an EasyDip Slide Staining jar containing 70% ethanol. Incubate for 5 min. Transfer the slides to a second staining jar containing fresh 70% ethanol and incubate for an additional 5 min.
34 Place the slides in an EasyDip Slide Staining jar containing 50% ethanol. Incubate for 5 min. Transfer the slides to a second staining jar containing fresh 50% ethanol and incubate for an additional 5 min.
35 Rinse the slides briefly in ddH_2_O for 1 min to complete rehydration.

#### Antigen Retrieval

**Figure 2:**
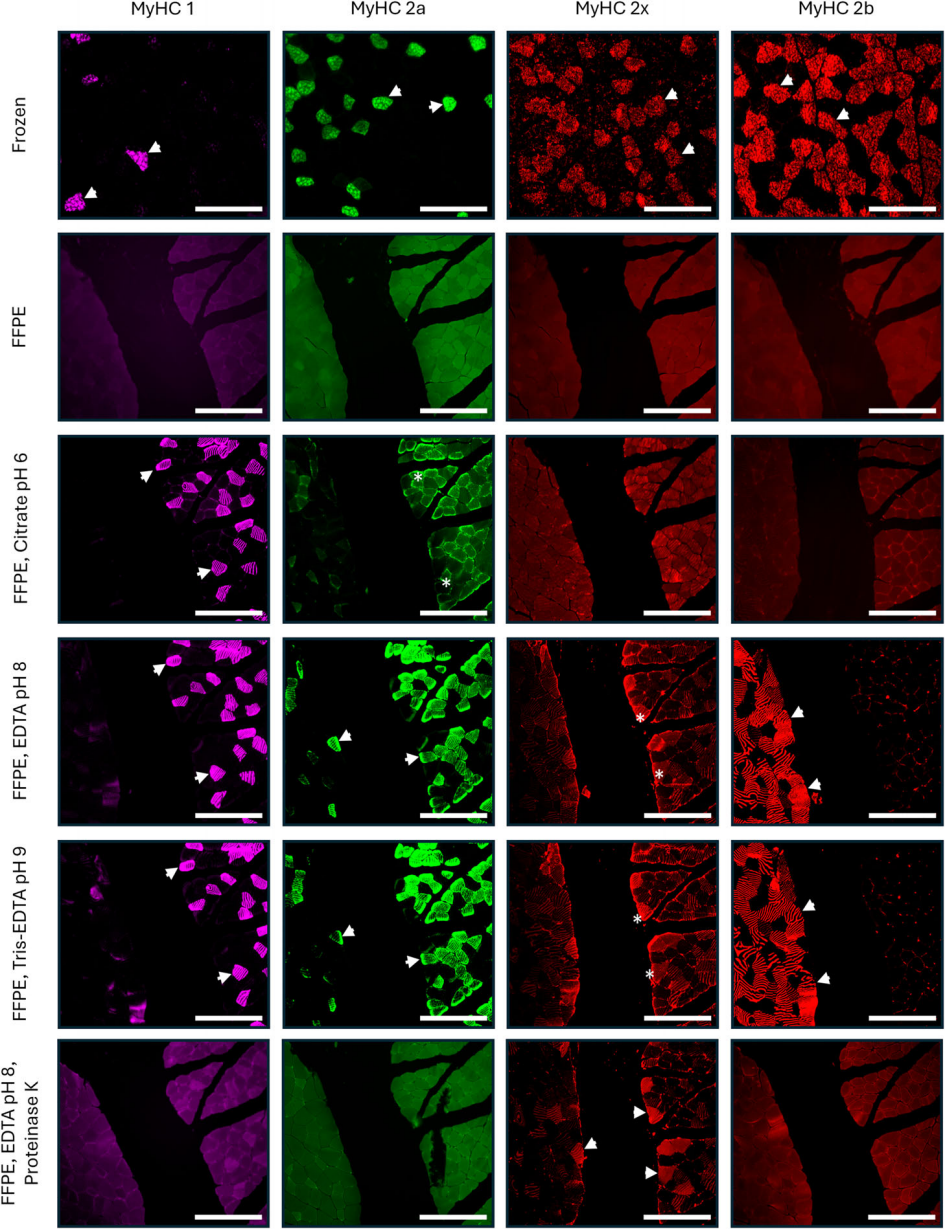
Effects of antigen retrieval procedures on MyHC immunolabeling. Row 1: Representative images of serial sections of fresh frozen quadriceps muscle preparations (conventional method) demonstrating immunoreactivity with antibodies to MyHC 1, MyHC 2a, MyHC 2x, and MyHC 2b. Row 2‐6: Sections of FFPE mouse muscle were stained with antibodies to MyHC 1, MyHC 2a, MyHC 2x, and MyHC 2b to evaluate the effectiveness of antigen unmasking using different antigen retrieval methods. Each image contains two muscles: plantaris on the left, soleus on the right, to include all fiber types in a single image. Row 2 shows that all MyHC antigens are masked on FFPE tissue preparations. *NOTE*: image brightness was enhanced for tissue visualization. Antigen retrieval methods tested on FFPE sections included HIAR using Sodium Citrate Buffer, pH 6.0 (Row 3), HIAR using EDTA, pH 8.0 (Row 4), HIAR using Tris‐EDTA, pH 9.0 (Row 5), and a combination of HIAR using EDTA, pH 8.0, followed by PIAR using Proteinase K digestion (Row 6). Immunostaining with each antibody was performed individually with antibodies against MyHC 1, MyHC 2a, MyHC 2x, and MyHC 2b. Retrieval with Tris‐EDTA, pH 9.0, and EDTA, pH 8.0, enhanced epitope accessibility for MyHC 1, MyHC 2a, and MyHC 2b, while proteinase K combined with HIAR improved staining for MyHC 2x. However, the combined HIAR and PIAR approach was not effective for labeling MyHC 1, MyHC 2a, and MyHC 2b. Overall, high‐pH antigen retrieval methods were most effective for unmasking MyHC 1, MyHC 2a, and MyHC 2b in FFPE muscle sections. Arrows indicate strongly labeled fibers. Asterisks indicate weakly labeled fibers. Scale bars = 200 µm.

36 Fill an Instant Pot (or comparable pressure cooker) with 500 ml ddH_2_O. Place a polypropylene Coplin staining jar filled with Tris‐EDTA buffer, pH 9.0, inside the pot.
37 Pre‐heat the Tris‐EDTA buffer, pH 9.0, during rehydration: On the Instant Pot, select the “Pressure Cook” button. Use the control dial on the Instant Pot to select the “Custom” program. Use the Control Dial to set Pressure to “High” and set the duration to “00:01” (1 min). Turn on the “Keep Warm” function. Start the Instant Pot program.
  The high‐pressure setting on the Instant Pot corresponds to a pressure between 10.2 and 11.6 psi and a temperature between 115°C and 118°C.
38 Once pre‐heated, carefully place the slides into the hot Tris‐EDTA buffer, pH 9.0, inside the polypropylene Coplin staining jar.
39 Begin antigen retrieval: On the Instant Pot, select the “Pressure Cook” button. Use the control dial on the Instant Pot to select the “Custom” program. Use the Control Dial to set pressure to “High” and set the duration time to “00:05” (5 min). Turn off the “Keep Warm” button. Start the Instant Pot program.
40 When the Instant Pot program is complete, allow pressure to release naturally. Remove the Coplin jar and let cool to RT for 30 min.
  Antigen retrieval methods are commonly used to restore epitope accessibility on FFPE sections. Different heat‐induced antigen retrieval (HIAR) buffers can favor different epitopes. Therefore, we empirically tested common HIAR buffers to determine which buffer provided the strongest reactivity for each MyHC isoform individually and to identify a common condition for multiplex IF MyHC isoform labeling (Fig. 2, see Table 1 for summary). Antibodies recognizing MyHC 1, MyHC 2a, MyHC 2x, and MyHC 2b were tested individually using the following methods: 1) No antigen retrieval; 2) HIAR with sodium citrate buffer, pH 6.0; 3) HIAR with EDTA buffer, pH 8.0; 4) HIAR with Tris‐EDTA buffer, pH 9.0; 5) HIAR with EDTA buffer, pH 8.0, followed by protease‐induced antigen retrieval (PIAR) using Proteinase K digestion. Immunofluorescence labeling of fresh‐frozen sections was performed to confirm immunoreactivity with antibodies to MyHC 1, MyHC 2a, MyHC 2x, and MyHC 2b, as reported elsewhere (Bean et al., 2025). Alkaline buffers, Tris‐EDTA (pH 9.0) and EDTA (pH 8.0), were best for enhancing epitope accessibility for MyHC 1, MyHC 2a, and MyHC 2b. However, all HIAR methods provided poor antigen unmasking for MyHC 2x. A prior report indicated that a combination of HIAR and enzymatic treatment with proteinase K was effective for unmasking the MyHC 2x antigen for immunohistochemistry in FFPE muscle sections (Ellefsen et al., 2014). Indeed, we also observed effective unmasking of MyHC 2x with both HIAR and proteinase K treatment. However, effective unmasking of MyHC 2x came at the expense of labeling of MyHC 1, 2a, and 2b, which were inhibited by proteinase K treatment. Thus, we conclude that MyHC 2x immunolabeling requires unique conditions (see Alternative Protocol 1) that are not compatible with multiplexing for other MyHC isoforms under the conditions that we tested.

**Table 1:** Antigen Retrieval and Immunolabeling Compatibility

		Antibody			
		MyHC1	MyHC2a	MyHC2×	MyHC2b
Antigen Retrieval Method	No Antigen Retrieval	No Signal	No Signal	No Signal	No Signal
	Sodium Citrate	Strong Signal	Weak Signal	No Signal	No Signal
	EDTA	Strong Signal	Strong Signal	Weak Signal	Strong Signal
	Tris‐EDTA	Strong Signal	Strong Signal	Weak Signal	Strong Signal
	EDTA + Proteinase K	No Signal	No Signal	Strong Signal	No Signal

#### Immunofluorescence Staining

41 Wash slides in 1× PBS for 5 min.
42 Use a hydrophobic barrier pen to carefully encircle the tissue sections on each slide.
43 Permeabilize tissue sections in 1× PBS containing 0.25% Triton X‐100 for 10 min at room temperature.
44 Block sections by incubating in Blocking Buffer for 30 min at room temperature in a humidified chamber.
45 Prepare primary antibodies against MyHC 1, MyHC 2a, and MyHC 2b isoforms and laminin by diluting in fresh Blocking Buffer (Table 2). Keep on ice.
  If applying this protocol to human muscle sections, exclude the antibody to MyHC 2b. Human muscle contains only type 1, 2a, and 2x fibers, whereas mouse muscle also contains a distinct type 2b fiber population. Consequently, while the MyHC 2b antibody is used to identify type 2b fibers in mouse sections, it should not be applied to human samples.

**Table 2:** Primary Antibodies

Clonality	Antibody Type	Target	Working concentration	Source	RRID
Monoclonal: Clone BA‐D5	Mouse isotype IgG2b	MyHC 1	1.2 µg/ml	DSHB	AB_2235587
Monoclonal: Clone SC‐71	Mouse isotype IgG1	MyHC 2a	5.58 µg/ml	DSHB	AB_2147165
Monoclonal: Clone BF‐F3	Mouse isotype IgM	MyHC 2b	0.85 µg/ml	DSHB	AB_2266724
Monoclonal: Clone 6H1	Mouse isotype IgM	MyHC2x	3.4 µg/ml	DSHB	AB_2314830
Polyclonal	Rabbit isotype IgG	Laminin	10 µg/ml	Millipore Sigma	AB_477163

46 Aspirate the blocking buffer from the slides and add the prepared primary antibody solution to completely cover the tissue sections. Incubate slides overnight at 4°C in a humidified chamber.
47 Wash slides in 1× PBS three times, 5 min each.
48 Quench autofluorescence. Incubate slides in TrueBlack solution for 30 s. *NOTE*: Do not use detergent in the buffer after applying TrueBlack.
  Optional step: TrueBlack autofluorescence quench is an optional step. However, we find that this step helps to reduce high autofluorescence levels known to occur in FFPE preparations.
49 Wash slides in 1× PBS three times, 5 min each.
50 Prepare the fluorophore‐conjugated secondary antibodies by diluting in 1× PBS (Table 3). Keep the antibody solution protected from light.
  If applying this protocol to human muscle sections, exclude goat anti‐mouse IgM Alexa Fluor 594.

**Table 3:** Secondary Antibodies

Secondary Antibody	Target	Working concentration	Source	RRID
Goat anti‐mouse IgG2b Alexa Fluor 647	Anti‐MyHC 1	2 µg/ml	Invitrogen	AB_2535811
Goat anti‐mouse IgG1 Alexa Fluor 488	Anti‐MyHC 2a	4 µg/ml	Invitrogen	AB_2535764
Goat anti‐mouse IgM Alexa Fluor 594	Anti‐MyHC 2b	2 µg/ml	Invitrogen	AB_2535713
Goat anti‐rabbit IgG Alexa Fluor 350	Anti‐Laminin	4 µg/ml	Invitrogen	AB_2534101

**Figure 3:**
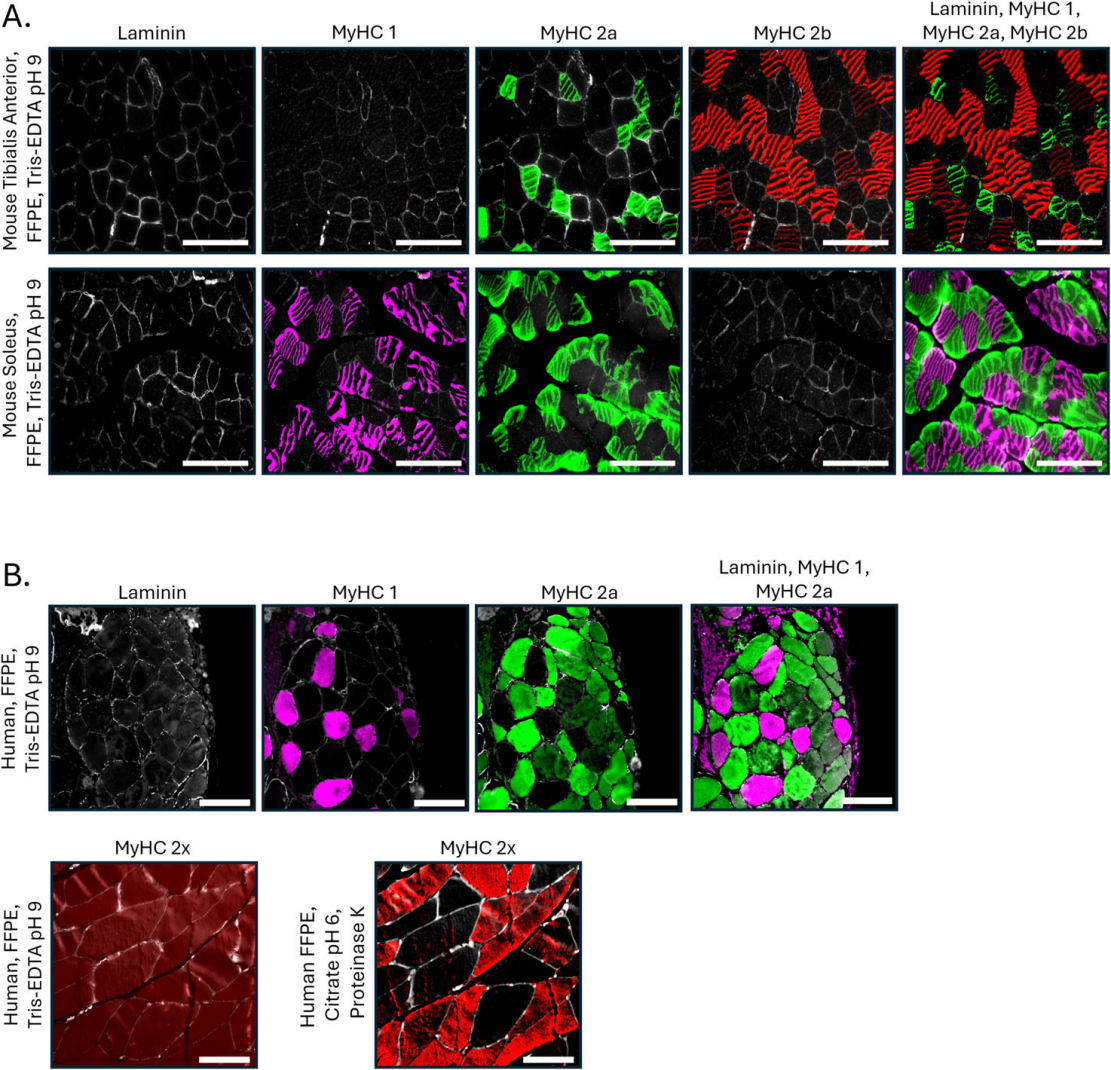
Immunofluorescence staining on FFPE muscle sections from mouse and human samples demonstrating the specificity of MyHC antibodies. (**A**) Serial sections were taken of the mouse TA (top) and soleus (middle) FFPE muscle, and the same field‐of‐view from each muscle was taken. Antigen retrieval was performed using Tris‐EDTA buffer, pH 9.0. Mouse sections were stained with anti‐MyHC 1 (magenta) and anti‐laminin (white), anti‐MyHC 2a (green) and anti‐laminin, anti‐MyHC 2b (red) and anti‐laminin, or with anti‐MyHC 1, anti‐MyHC 2a, anti‐MyHC 2b, and anti‐laminin combined. MyHC 2x fibers = unlabeled (black). Scale bars = 100 µm. (**B**) Human FFPE biopsies from the right arm were provided by the Biospecimen Collection and Banking Core at the IU Simon Comprehensive Cancer Center, sectioned, and images from the same field‐of‐view were taken. Antigen retrieval was performed using Tris‐EDTA, pH 9.0, and sections were stained with anti‐MyHC 1 (magenta) and anti‐laminin (white), anti‐MyHC 2a (green) and laminin, or anti‐MyHC 1, anti‐MyHC 2a, and anti‐laminin combined (top panel). Anti‐MyHC 2x immunolabeling was tested using two antigen retrieval methods: 1) Tris‐EDTA, pH 9.0, HIAR; 2) sodium citrate, pH 6.0, HIAR followed by proteinase K PIAR (bottom panel). Tris‐EDTA, pH 9.0, HIAR failed to produce consistent labeling. Sodium citrate, pH 6.0, HIAR followed by proteinase K PIAR enabled successful detection of type 2x fibers in human FFPE muscle, as reported elsewhere (**Ellefsen et al.,** 2014). Scale bars = 100 µm.

51 Apply the prepared secondary antibody solution to fully cover each tissue section. Incubate the slides for 1 hr at RT in the dark, inside a humidified chamber.
52 Wash slides in 1× PBS three times, 5 min each.
53 Mount slides with ProLong Gold Antifade Mountant. Carefully apply coverslips to avoid bubbles.
  Use DAPI‐free mountant to prevent spectral overlap with Laminin staining.
54 Visualize the stained sections using an epifluorescence or confocal microscope, selecting filter sets appropriate for each fluorophore conjugated to the secondary antibodies.
  For mouse sections, laminin will appear in the DAPI channel, type I fibers in the Cy5 channel, type 2a fibers in the GFP channel, and type 2b fibers in the Texas Red channel. Type 2x fibers will remain unlabeled and appear black (Fig. 3A). For human sections, laminin will appear in the DAPI channel, type I fibers in the Cy5 channel, and type 2a fibers in the GFP channel (Fig. 3B).

## Alternate Protocol 1 IMMUNOFLUORESCENCE FOR MyHC 2x LABELING IN FFPE SKELETAL MUSCLE TISSUES

This protocol focuses on the specific detection of type 2x muscle fibers in FFPE skeletal muscle using an antibody directed against the MyHC 2x isoform. Because the antibody for MyHC 2x requires distinct antigen retrieval conditions and is not compatible with the multiplex panel in Basic Protocol, this method is optimized for specific labeling of type 2x fibers. The procedure follows similar steps of deparaffinization and rehydration utilized in the Basic Protocol, but incorporates an antigen‐retrieval method tailored for the MyHC 2x antibody. This protocol results in specific labeling of type 2x fibers, enabling focused analysis of this fiber subtype.

### [Additional] Materials

EDTA Buffer, pH 8.0 (see recipe)
Sodium Citrate Buffer, pH 6.0 (see recipe)
Working Proteinase K Solution (see recipe)
Mouse IgM anti‐Rabbit Myosin Heavy Chain Type 2× (Developmental Studies Hybridoma Bank, cat. no. 6H1)

Hybridization Oven (Robbins Scientific, cat. no. 1040‐60‐1AG or equivalent)

#### Formalin Fixation and Paraffin Embedding

1 Perform formalin fixation and paraffin embedding according to Basic Protocol steps 1–8.

#### Deparaffinization and Rehydration

2 Perform deparaffinization and rehydration according to Basic Protocol steps 9–14.

#### Antigen Retrieval

3 Fill an Instant Pot (or comparable pressure cooker) with 500 ml ddH_2_O. Place a polypropylene Coplin staining jar filled with EDTA buffer, pH 8.0, inside the pot.
  If applying this protocol to human sections, use sodium citrate buffer, pH 6.0, instead of EDTA buffer, pH 8.0, for HIAR.
4 Pre‐heat the EDTA buffer, pH 8.0, during rehydration steps: On the Instant Pot, select the “Pressure Cook” button. Use the control dial on the Instant Pot to select the “Custom” program. Use the Control Dial to set Pressure to “High” and set the duration to “00:01”. Turn on the “Keep Warm” function. Start the Instant Pot program.
  If applying this protocol to human sections, use sodium citrate buffer, pH 6.0, instead of EDTA buffer, pH 8.0, for HIAR.
5 Once pre‐heated, carefully place the slides into the hot EDTA buffer, pH 8.0, inside the polypropylene Coplin staining jar.
  If applying this protocol to human sections, use sodium citrate buffer, pH 6.0, instead of EDTA buffer, pH 8.0, for HIAR.
6 Begin antigen retrieval: On the Instant Pot, select the “Pressure Cook” button. Use the control dial on the Instant Pot to select the “Custom” program. Use the Control Dial to set pressure to “High” and set the duration to “00:05” (5 min). Turn off the “Keep Warm” button. Start the Instant Pot program.
7 When the Instant Pot program is complete, allow the pressure to release naturally. Remove the Coplin jar and let cool to RT for 30 min.
8 Wash slides in 1× PBS for 5 min.
9 Use a hydrophobic barrier pen to carefully encircle the tissue sections on each slide.
10 Apply enough Working Proteinase K Solution to fully cover each tissue section. Incubate the sections at 37°C in a hybridization oven for 10 min.

#### Immunofluorescence Staining

11 Wash slides in 1× PBS three times, 2 min each.
12 Permeabilize tissue sections in 1× PBS containing 0.25% Triton X‐100 for 10 min at RT.
13 Block sections by incubating in Blocking Buffer for 30 min at RT in a humidified chamber.
14 Prepare the primary antibody against MyHC 2x by diluting in fresh Blocking Buffer (Table 2). Keep on ice.
15 Aspirate the blocking buffer from the slides and add the prepared primary antibody solution to completely cover the tissue sections. Incubate slides overnight at 4°C in a humidified chamber.
16 Wash slides in 1× PBS three times, 5 min each.
17 Quench autofluorescence: incubate slides in TrueBlack solution for 30 s.
18 Wash slides in 1× PBS three times, 5 min each.
19 Prepare goat anti‐mouse IgM Alexa Fluor 594 secondary antibody by diluting in 1× PBS (Table 3). Protect from light.
20 Apply the prepared secondary antibody solution to fully cover each tissue section. Incubate the slides for 1 hr at RT in the dark, inside a humidified chamber.
21 Wash slides in 1× PBS three times, 5 min each.
22 Mount slides with ProLong Gold Antifade Mountant. Carefully apply coverslips to avoid bubbles.
23 Visualize the stained sections using an epifluorescence or confocal microscope, selecting the Texas Red filter.
  Type 2x fibers will appear in the Texas Red channel for both mouse (Fig. 2) and human (Fig. 3B) sections.

## Alternate Protocol 2 MULTIPLEX IMMUNOFLUORESCENCE FOR MyHC LABELING IN FFPE WHOLE HINDLIMB SECTIONS

This protocol details processing steps for preparing the intact mouse lower hindlimb for multiplex IF analysis, including perfusion fixation and bone demineralization. Following processing, FFPE sections are subjected to multiplex IF labeling using antibodies against MyHC. This approach preserves the spatial relationships among muscle and associated tissues, allowing comprehensive fiber type and morphological analysis across anatomically distinct muscles within a single tissue section.

### [Additional] Materials

1.2% 2,2,2‐Triboromoethanol (see recipe)
Demineralization buffer (see recipe)

25‐Gauge needle (BD Bioscience cat. no. 305122 or equivalent)
10‐ml Syringe (Fisherbrand cat. no. 14‐955‐459 or equivalent)
0.2‐µm solvent syringe filter (Sartorius cat. no. 17845ACK or equivalent)
Razor blades (Fisherbrand cat. no. 12640 or equivalent)
50‐ml Conical Tube (Falcon cat. no. 1443222 or equivalent)
Rocker (Boekel Scientific cat. no. 260350 or equivalent)
*All animal procedures described in this protocol were performed in accordance with guidelines approved by IACUC at Indiana University School of Medicine (protocol #22045, approval date: 3 August 2022). Researchers intending to use this protocol must obtain prior approval from IACUC or their equivalent regulatory body before performing the following procedures*.

#### Perfusion and Fixation

**Figure 4:**
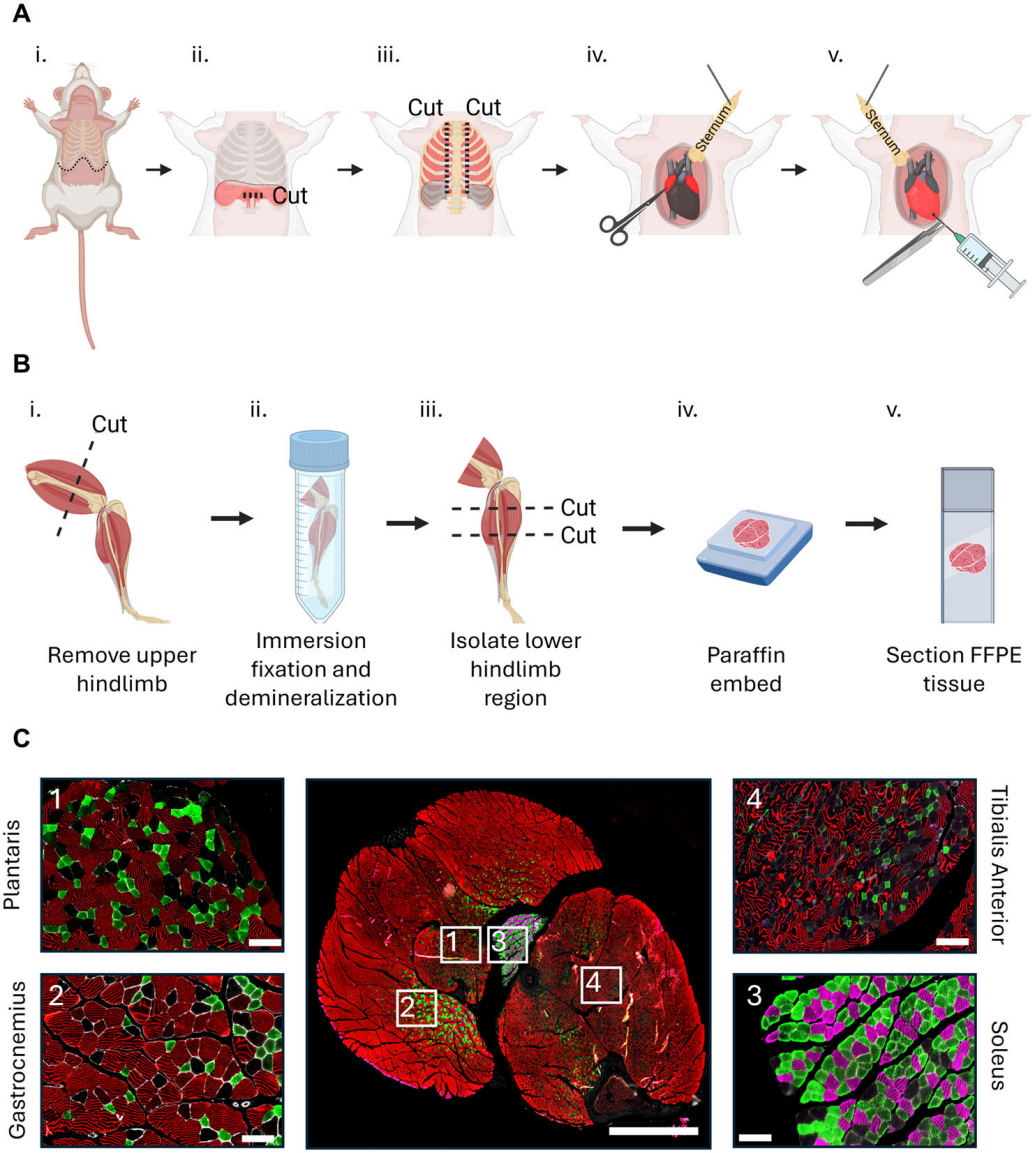
Whole hindlimb processing and immunofluorescence fiber type staining. (**A**) Schematic overview of a mouse perfusion in preparation for whole hindlimb isolation. Steps include: (i) Cutting directly inferior to xiphoid process and along the rib cage towards the spine to expose the abdominal cavity, (ii) Making a small incision in the diaphragm, (iii) Cutting along both sides of the sternum to expose the heart, (iv) Pinning the sternum over the left shoulder and making an incision in the right atrium, (v) Pinning the sternum over the right shoulder and perfusing the mouse. (**B**) Schematic overview of the experimental workflow for isolating the whole mouse hindlimb for FFPE tissue sectioning. Steps include: (i) Cutting and removing the upper hindlimb (proximal to the knee joint) to isolate the lower hindlimb, (ii) Immersion fixation for 24 hr followed by demineralization for 14 days, (iii) Performing two cuts on the lower hindlimb to isolate region of interest, recommended to cut at the tibial tuberosity and 5 mm distal to the tibial tuberosity, (iv) Dehydration and paraffin embedding of tissue, (vi) Sectioning of FFPE tissue in preparation for immunofluorescence staining. C: Representative immunofluorescence staining of an intact mouse lower hindlimb. The central image shows the whole hindlimb with numbered boxed regions indicating areas highlighted in the surrounding panels. Panels 1–4 display higher‐magnification views of different muscles: (1) plantaris, (2) gastrocnemius, (3) soleus, and (4) TA. Fiber type labeling and tissue architecture are preserved across the section, demonstrating the capability of this protocol to visualize multiple muscles within the intact hindlimb. Montage bar = 2000 µm. Insert bar = 500 µm.

1 Aliquot 45 ml of cold 1× PBS into a 50‐ml conical tube. Fill three, 10‐ml syringes with 10 ml of 1× PBS and attach a 25‐gauge needle. Keep cold until perfusion.
2 Aliquot 45 ml cold neutral buffered formalin into a 50‐ml conical tube. Fill three, 10‐ml syringes with 10 ml neutral buffered formalin and attach a 25‐gauge needle. Keep cold until perfusion.
3 Anesthesia: Anesthetize the mouse by injecting with 1.2% 2,2,2‐Triboromoethanol (TBE) solution (or lab's preferred anesthetic) to a dosage of 250 mg/kg or to effect.
4 After the mouse is anesthetized, confirm lack of response of the pedal withdrawal reflex. Briefly, firmly perform hindlimb toe pinch with forceps to confirm the surgical plane. If the mouse is unresponsive, lay the mouse supine onto a dissection board.
  If the mouse is still responsive, administer additional anesthetic and repeat to effect.
5 Saturate the mouse with 70% ethanol. Use forceps to pick up the skin around the inferior portion of the sternum and make an excision with scissors.
6 Cut directly inferior to the xiphoid process of the sternum and along the rib cage towards the spine, exposing the abdominal cavity (Fig. 4Ai). Make a small incision in the diaphragm directly underneath the sternum (Fig. 4Aii). Using this incision, use blunt scissors to cut along both sides of the sternum to open the thoracic cavity and expose the heart (Fig. 4Aiii).

7 Make an incision on the right atrium using scissors so that blood and perfusate can drain. Pinning the sternum over the left shoulder will improve access to the right atrium (Fig. 4Aiv). Place pin with sternum over the right shoulder to provide ample exposure to the left ventricle. Use blunt‐end forceps to gently hold the heart, then pierce the left ventricle with the needle of the perfusion syringe containing cold 1× PBS (Fig. 4Av). Once the needle is in the left ventricle, use forceps to stabilize the needle to prevent piercing of the left atrium during perfusion.
8 Perfuse the mouse with 30 ml cold 1× PBS at a rate of 10 ml/min. Clear effluent indicates the blood is flushed.
9 Next, using the 10‐ml syringes containing neutral buffered formalin, insert the needle back into the same puncture site. Perfuse the mouse with 30 ml cold neutral buffered formalin.
  Tissues should become rigid after the perfusion is complete. Perfusion is performed to help maintain tissue morphology during paraffin embedding. Immersion fixation of the hindlimb is an acceptable alternative; however, anecdotally, we find the quality of the tissue morphology to be diminished.
10 Following perfusion, dissect the lower hindlimb. Use blunt scissors to cut proximal to the knee joint on the femur (Fig. 4Ai). Remove any excess hamstrings adjacent to the gastrocnemius muscle of the lower hindlimb.
  We recommend making the cut proximal to the knee and keeping the foot attached to preserve the tendons of the muscles of the lower hindlimb. Severing these tendons (e.g., the Achilles tendon) disrupts the mechanical connection of the lower hindlimb muscles, leading to loss of resting tension and optimal muscle length, affecting tissue morphology.
11 Place the lower hindlimb into a 50‐ml conical tube containing 30 ml neutral buffered formalin and incubate 24 hr at 4°C with gentle agitation on a rocker.

#### Demineralization

12 Transfer the lower hindlimb to a 50‐ml conical tube containing 30 ml demineralization buffer.
13 Incubate the lower hindlimb in demineralization buffer for 14 days at 4°C with gentle agitation on a rocker. Change the demineralization buffer every 3–4 days (Fig. 4Aii).
  Validate demineralization is complete after 14 days by inserting a 25‐gauge needle into a region of the bone that will not be used for histology. If demineralization is complete, the pin should insert smoothly into the bone with little resistance. If demineralization is not complete, keep the lower hindlimb in demineralization buffer until a pin can be inserted smoothly into the bone.
14 After demineralization is complete, wash specimens in running ddH_2_O for 60 min.
15 Transfer the lower hindlimb to a 50‐ml conical tube containing 30 ml of 70% ethanol. Store at 4°C for at least 24 hr.
16 Remove the lower hindlimb from ethanol. Using a razor blade, make two transverse cuts through the mid‐belly of the muscles across the lower hindlimb (Fig. 4Aiii).
  We recommend using anatomical landmarks to ensure consistency across samples. Make the proximal cut at the tibial tuberosity. Perform a distal cut ∼5 mm distal to the proximal cut.
17 Perform dehydration, paraffin embedding, and sectioning according to your lab's or histology facility's standard protocol. Cut sections at 5‐µm thickness.

#### Deparaffinization and Rehydration

18 Perform deparaffinization and rehydration according to Basic Protocol steps 9–14.

#### Immunofluorescence Staining

19 Perform immunofluorescence staining according to Basic Protocol steps 15–33.

## Reagents and Solutions

### Blocking Buffer

250 µl Normal Goat Serum (5% v/v final; Invitrogen, cat. no. 31872)
4.75 ml PBS 0.25% Triton‐X 100 (95% v/v final; Millipore Sigma, cat. no. EM‐TX1568‐1)
Prepare fresh and use on the day of the experiment

### Demineralization Buffer

500 ml ddH_2_O
10.0 g NaOH (10% w/v final; Fisher Scientific, cat. no. S318500)
100.0 g Ethylenediaminetetraacetic Acid, Disodium Salt Dihydrate (342 mM final; Fisher Chemical, cat. no. S311)
Add ddH_2_O to 700 ml total volume
Add 300 ml of 10% neutral buffer formalin (30% v/v final; Fisherbrand, cat. no. 11002205)
Adjust to pH 7.4 with 1 M NaOH
Store at 4°C for up to 3 months

### EDTA Buffer, pH 8.0

800 ml ddH_2_O
0.372 g Ethylenediaminetetraacetic Acid, Disodium Salt Dihydrate (1 mM final; Fisher Chemical, cat. no. S311)
Add ddH_2_O to 1 L total volume
Adjust to pH 8.0 with 1 M NaOH
Store at RT for up to 3 months

### Ethanol, 95%

950 ml of 200‐Proof Ethanol (95% v/v final; Decon Labs, cat. no. 04355223 or equivalent)
50 ml ddH_2_O

### Ethanol, 70%

700 ml of 200‐Proof Ethanol (70% v/v final; Decon Labs, cat. no. 04355223 or equivalent)
300 ml ddH_2_O

### Ethanol, 50%

500 ml of 200‐Proof Ethanol (50% v/v final; Decon Labs, cat. no. 04355223 or equivalent)
500 ml ddH_2_O

### Phosphate‐Buffered Saline (PBS), 10×

800 ml ddH_2_O
80 g NaCl (137 mM final; Fisher Bioreagents, cat. no. BP358 or equivalent)
2 g KCl (2.7 mM final; Fisher Bioreagents, cat. no. P217 or equivalent)
14.2 g Na_2_HPO_4_ Dibasic Anhydrous (10 mM final; Fisher Bioreagents, cat. no. S374 or equivalent)
2.4 g KH_2_PO_4_ Monobasic (1.8 mM final; Fisher Bioreagents, cat. no. BP362 or equivalent)
Add ddH_2_O to 1 L total volume
Adjust to pH 7.4 with 1 M NaOH
Store at room temperature for up to 6 months

### PBS, 1×

900 ml ddH_2_O
100 ml of 10× PBS (10% v/v final)
Store at room temperature for up to 3 months

### PBS, 1×, containing 0.25% Triton X‐100

897.5 ml ddH_2_O
100 ml of 10× PBS (10% v/v final)
2.5 ml Triton X‐100 (0.25% v/v final; Millipore Sigma, cat. no. EM‐TX1568‐1)
Store at room temperature for up to 3 months

### Sodium Citrate Buffer, pH 6.0

800 ml ddH_2_O
2.94 g Sodium Citrate Tribasic Dihydrate (10 mM final; Sigma Aldrich, cat. no. S4641)
0.5 ml Tween‐20 (0.05% v/v final; Fisher Bioreagents, cat. no. BP337)
Add ddH_2_O to 1 L total volume
Adjust to pH 6.0 with 1M HCl
Store at 4°C for up to 6 months

### 2,2,2‐Triboromoethanol (TBE), 50%

0.5 g of 2,2,2‐Tribromoethanol (50% w/v final; Thermo Chemical, cat. no. AC421430100)
1 ml of 2‐methyl‐2‐butanol Reagent Plus, 99% (Sigma Aldrich, cat. no. 152463)
Dissolve by heating to 50°C overnight in a chemical fume hood. Protect from light
Store at 4°C for up to 6 months

### TBE, 1.2%

243 µl of 50% TBE (1.2% w/v final)
10 ml ddH_2_O
Sterile filter using 0.2‐µm solvent syringe filter
Store at 4°C protected from light for up to 2 weeks

### TE‐CaCl2 Buffer

70 ml ddH_2_O
0.605 g Tris Base (50 mM final; Fisher Bioreagents, cat. no. BP152)
0.074 g CaCl_2_ Dihydrate (5 mM final; Sigma Aldrich, cat. no. C7902)
0.037 g Ethylenediaminetetraacetic Acid, Disodium Salt Dihydrate (1 mM final; Fisher Chemical, cat. no. S311)
0.5 ml Triton X‐100 (0.5% v/v final; Millipore Sigma, cat. no. EM‐TX1568‐1)
Add ddH_2_O to 100 ml total volume
Adjust to pH 8.0 with 1 M NaOH
Store at room temperature for up to 3 months

### Tris‐EDTA Buffer, pH 9.0

800 ml ddH_2_O
1.21 g Tris Base (10 mM final; Fisher Bioreagents, cat. no. BP152)
0.372 g Ethylenediaminetetraacetic Acid, Disodium Salt Dihydrate (1 mM final; Fisher Chemical, cat. no. S311)
0.5 ml Tween‐20 (0.05% v/v final; Fisher Bioreagents, cat. no. BP337)
Add ddH_2_O to 1 L total volume
Adjust to pH 9.0 with 1 M NaOH
Store at room temperature for up to 3 months

### Working Proteinase K Solution

999 µl TE‐CaCl2 Buffer (99% v/v)
1 µl Proteinase K (1% v/v; Qiagen, cat. no. 19133)
Prepare fresh and use on the day of the experiment

## COMMENTARY

### Critical Parameters

Critical parameters for successful multiplex immunofluorescence labeling of MyHC isoforms on FFPE muscle sections include careful optimization of antigen retrieval conditions. Excessive heat or prolonged antigen retrieval can cause tissue damage and adversely affect antibody binding (Kiernan, 2015). To mitigate this, users can adjust pressure settings or shorten heat exposure time during antigen retrieval if signs of tissue damage, such as tearing, folding, or loss of morphology, are observed. Conversely, if antibody labeling is weak or absent, increasing the pressure or duration of antigen retrieval may enhance antigen unmasking and improve staining intensity (Grillo et al., 2017). Different epitopes require distinct antigen retrieval conditions. Specifically, Basic Protocol (for MyHC 1, 2a, and 2b) utilizes HIAR with Tris‐EDTA buffer, pH 9.0. In contrast, Alternate Protocol 1 (for MyHC 2x) utilizes HIAR with EDTA buffer, pH 8.0, for mouse sections or Sodium Citrate buffer, pH 6.0, for human sections, followed by proteinase K treatment. Various antigen retrieval buffers, including sodium citrate, Tris‐EDTA, EDTA alone, and EDTA followed by proteinase K digestion, were tested for each antibody. Some were suitable for certain antibodies but not others (Fig. 2, Table 1).

Autofluorescence, particularly in the red fluorescence channels, can interfere with the identification of unlabeled type 2x fibers in multiplex staining. The application of TrueBlack reagent is highly recommended to quench autofluorescence and clearly distinguish type 2x fibers, which appear as unlabeled dark areas.

The type 2x fiber detection protocol (Alternate Protocol 1) typically results in weaker antibody labeling compared to other MyHC isoforms. It requires careful adjustment of antibody concentration and imaging settings to enhance signal detection. Importantly, human muscle contains only type 1, 2a, and 2x fibers, whereas mouse muscle also contains a distinct type 2b fiber population. Consequently, while the MyHC 2b antibody is routinely used to identify type 2b fibers in mouse sections, it should not be applied to human samples, where no equivalent type 2b fibers exist (Smerdu et al., 1994). It is also important to note that in human muscle, the MyHC 2a monoclonal SC‐71 antibody has been reported to cross‐react with both type 2a and type 2x fibers, which necessitates careful interpretation when analyzing human samples (Murach et al., 2019). However, reductions in the concentration of the SC‐71 antibody can reduce its cross‐reactivity (Bloemberg & Quadrilatero, 2012).

### Troubleshooting Table

Issues that can be encountered in these IF protocols, their potential causes, and recommended solutions are summarized in Table 4. This guide is intended to help users identify and resolve problems to achieve optimal results.

**Table 4:** Troubleshooting Guide for MyHC IF

Problem	Possible cause	Solution
Weak or no antibody signal	Inadequate antigen retrieval	Optimize antigen retrieval heat/time or buffer; see Table 1 for buffer suitability
Tissue damage or loss of morphology	Overheating or prolonged antigen retrieval	Reduce heat/time during antigen retrieval step
High background or autofluorescence	Insufficient quenching of tissue autofluorescence	Apply TrueBlack reagent prior to imaging
Poor labeling of type 2x fibers	Incorrect HIAR antigen retrieval buffer	For mouse sections, use EDTA buffer, pH 8.0, for HIAR. For human sections, use sodium citrate buffer, pH 6.0

### Understanding Results

Skeletal muscles are composed of diverse fiber types whose proportions vary depending on the muscle's functional role. In mice, predominantly slow‐twitch muscles, such as the soleus, contain a higher percentage of type 1 and 2a fibers, which are adapted for endurance and oxidative metabolism. These fibers typically have smaller cross‐sectional areas in mice and higher concentrations of oxidative enzymes. In contrast, fast‐twitch muscles, such as the TA and extensor digitorum longus, have a greater abundance of type 2x and 2b fibers, which support forceful contractions but fatigue more quickly, and these fibers generally exhibit larger cross‐sectional areas in mice (Augusto et al., 2017; Burkholder et al., 1994; Pette & Staron, 2000). In humans, cross‐sectional area is not correlated with fiber type (Fournier et al., 2022; O'Reilly et al., 2021). Moreover, some muscles demonstrate regional heterogeneity, with fiber type composition varying between different areas within the same muscle. Using a multiplex immunofluorescence protocol, these distinctions between muscles manifest as clear differences in fiber type labeling patterns. The mouse soleus shows a relatively uniform distribution of type 1 and type 2a fibers with minimal labeling of type 2x and 2b fibers (Fig. 4). In contrast, the mouse TA displays a more heterogeneous mosaic of fiber types (Fig. 4). Changes in fiber type distribution can be indicative of underlying neuropathological or physiological processes. For example, in humans, aging is associated with fiber type‐specific changes, such as the preferential atrophy and loss of type 2 fibers and increased proportion of type 1 fibers (Evans & Lexell, 1995). Furthermore, exercise (or lack of) can induce adaptive remodeling of fiber types, as human studies showed that endurance training increased the proportion of type 1 fibers, resistance training increased the proportion of type 2a fibers, and a sedentary lifestyle increased the proportion of type 2a and 2x fibers (Plotkin et al., 2021; Wilson et al., 2012). In addition to age‐ and exercise‐related changes, pathological changes can affect fiber type distribution. For example, muscle fiber denervation can lead to fiber type grouping, a process in which fibers of the same type cluster together rather than appearing as the normal interspersed mosaic pattern. This occurs when surviving motor neurons reinnervate neighboring denervated fibers, producing clusters of fibers of the same fiber type (Dedkov et al., 2003; Patterson et al., 2006). The IF protocols provided can help determine changes in fiber type distribution and provide insights into physiological and pathological muscle adaptations.

Analysis of the intact mouse lower hindlimb reveals spatial heterogeneity beyond individual muscles. For example, regions of the gastrocnemius muscle adjacent to the anterior compartment muscles display a higher proportion of type 2a and 2x fibers compared to more posterior areas (Fig. 4). Such regional differences within and between muscles emphasize the advantage of the multiplex approach on whole lower hindlimb sections, enabling comprehensive morphological analysis while preserving native tissue architecture. Additionally, fiber type analysis across the intact mouse lower hindlimb is particularly well suited for quantifying myofiber loss or hyperplasia. By capturing the entire lower hindlimb within a single preparation, this approach enables simultaneous assessment of fiber type‐specific changes in number, as well as size and distribution, across functionally distinct muscles.

### Time Considerations

#### Basic Protocol

Formalin Fixation and Paraffin Embedding: ∼3 days

Deparaffinization and Rehydration: ∼1.5 hr

Antigen Retrieval: ∼1 hr

Immunofluorescence Staining: 1 day, 3 hr

#### Alternate Protocol 1

Formalin Fixation and Paraffin Embedding: ∼3 days

Deparaffinization and Rehydration: ∼1.5 hr

Antigen Retrieval: ∼1 hr

Immunofluorescence Staining: 1 day, 4 hr

#### Alternate Protocol 2

Perfusion and Fixation: ∼1 day, 1 hr

Demineralization: ∼16 days

Deparaffinization and Rehydration: ∼1.5 hr

Antigen Retrieval: ∼1 hr

Immunofluorescence Staining: 1 day, 3 hr

### Author Contributions

**Connor Thomas**: Conceptualization; data curation; investigation; methodology; validation; writing—original draft; writing—review and editing. **Lainey Hibbard**: Conceptualization; methodology; writing—review and editing. **Kenneth White**: Conceptualization; methodology; writing—review and editing. **Steven Welc**: Conceptualization; funding acquisition, methodology; project administration; resources; supervision; validation; writing—review and editing.

### Conflict of Interest

There are no financial or personal relationships that present a conflict of interest.

## Data Availability

Data sharing not applicable to this article, as no datasets were generated or analyzed during the current study
